# Studying the Association of TKS4 and CD2AP Scaffold Proteins and Their Implications in the Partial Epithelial–Mesenchymal Transition (EMT) Process

**DOI:** 10.3390/ijms242015136

**Published:** 2023-10-13

**Authors:** Anita Kurilla, Loretta László, Tamás Takács, Álmos Tilajka, Laura Lukács, Julianna Novák, Rita Pancsa, László Buday, Virág Vas

**Affiliations:** 1 Institute of Enzymology, Research Centre for Natural Sciences, 1117 Budapest, Hungary; 2 Doctoral School of Biology, Institute of Biology, ELTE Eötvös Loránd University, 1117 Budapest, Hungary; 3 Department of Molecular Biology, Semmelweis University, 1094 Budapest, Hungary

**Keywords:** scaffold proteins, CD2AP, TKS4, protein–protein interaction, epithelial to mesenchymal transition, colon cancer

## Abstract

Colon cancer is a leading cause of death worldwide. Identification of new molecular factors governing the invasiveness of colon cancer holds promise in developing screening and targeted therapeutic methods. The Tyrosine Kinase Substrate with four SH3 domains (TKS4) and the CD2-associated protein (CD2AP) have previously been linked to dynamic actin assembly related processes and cancer cell migration, although their co-instructive role during tumor formation remained unknown. Therefore, this study was designed to investigate the TKS4-CD2AP interaction and study the interdependent effect of TKS4/CD2AP on oncogenic events. We identified CD2AP as a novel TKS4 interacting partner via co-immunoprecipitation-mass spectrometry methods. The interaction was validated via Western blot (WB), immunocytochemistry (ICC) and proximity ligation assay (PLA). The binding motif of CD2AP was explored via peptide microarray. To uncover the possible cooperative effects of TKS4 and CD2AP in cell movement and in epithelial-mesenchymal transition (EMT), we performed gene silencing and overexpressing experiments. Our results showed that TKS4 and CD2AP form a scaffolding protein complex and that they can regulate migration and EMT-related pathways in HCT116 colon cancer cells. This is the first study demonstrating the TKS4-CD2AP protein–protein interaction in vitro, their co-localization in intact cells, and their potential interdependent effects on partial-EMT in colon cancer.

## 1. Introduction

Several molecular events are known to drive cancer formation, including the accumulation of oncogenic mutations, development of drug resistance, and initiation of the cellular invasion cascade, leading to metastasis [1]. However, most of the molecular mechanisms underlying oncogenesis are not yet fully understood. The epithelial–mesenchymal transition (EMT) process, during which epithelial cells transform into the mesenchymal state, is the central regulator of metastasis [2]. Although EMT has been generally thought of as a binary process, it has recently been proven that it is instead a gradual transformation. In this spectrum, EMT includes an intermediate partial EMT state in which cells maintain both epithelial and mesenchymal phenotypes, implying that co-expression of epithelial and mesenchymal markers can be observed [3]. It is known that tumor cells can be heterogenous with epithelial, mesenchymal, and hybrid E/M (epithelial/mesenchymal) phenotypes simultaneously. Likewise, tumors with a partial EMT phenotype can also show intra-tumoral heterogeneity and cancer cells with different degrees of epithelial–mesenchymal plasticity. The partial-EMT process includes multiple steps, including early, intermediate, and late states that cover the spectrum from an epithelial to a completely mesenchymal phenotype [4]. Furthermore, partial-EMT has high clinical significance as it was associated with poorer prognosis and higher metastatic risk [5,6]. Presently, there is no clear consensus in defining the partial-EMT phenotype; however, co-expression of different epithelial and mesenchymal markers is considered an intermediate cell state [7].

**Figure 1:**
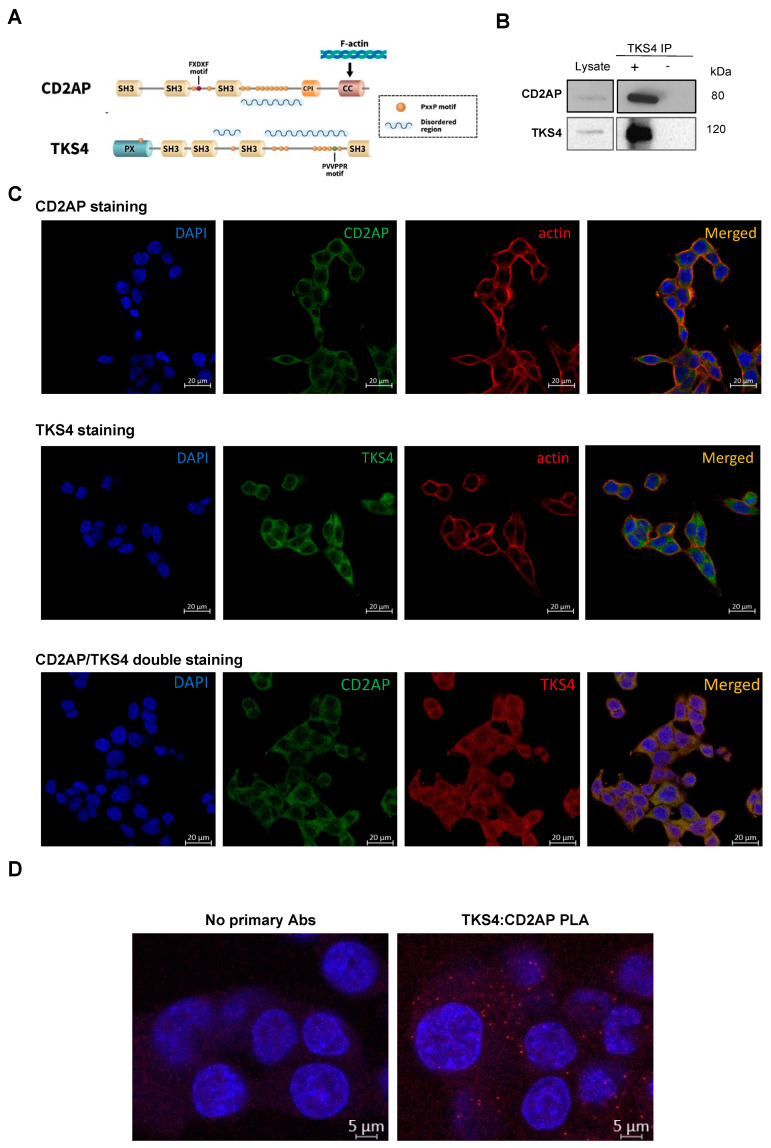
A combination of co-IP and MS was used to identify the CD2AP and TKS4 interaction, and the validation of CD2AP-TKS4 interaction via IP-WB, DuoLink assay and peptide array. (**A**) Schematic representation of CD2AP and TKS4 proteins highlighting the potential (proline-rich) interacting motifs in Tks4 molecule (PVVPPR). Domain names in the proteins are the following: CPI: capping protein interaction motifs, CC: coiled-coil region, SH3: SRC homology 3 domains, PX: phosphoinositide-binding domain. (**B**) Validation of CD2AP as a TKS4-interacting partner via IP-WB in HCT116 cells. The blot image is labeled as follows: Lysate sample means that the whole HCT116 cell lysates were loaded as positive control of the anti-CD2AP and the anti-TKS4 antibody staining; “+” and “−” *TKS4 IP* means that HCT116 cell lysates were immunoprecipitated with or without the anti-TKS4 antibody along with Sepharose-A. (**C**) ICC of single and double staining for CD2AP (Alexa 488 (green)) and TKS4 (Alexa 546 (red)) of HCT116 cells. The colocalization analysis of the two proteins showed overlap as orange fluorescent signals and have the following Pearson’s coefficients: in case of CD2AP/actin colocalization *p* = 0.762, in case of TKS4/actin colocalization *p* = 0.717, in case of CD2AP/TKS4 colocalization *p* = 0.817. (**D**) Detecting the CD2AP-TKS4 interaction in HCT116 cells via DuoLink PLA counterstained with DAPI. In control samples (stained with IgG_Rabbit_ and IgG_Mouse_ antibodies) no fluorescence signal was generated. In anti-TKS4 and anti-CD2AP antibodies-stained samples, the two DPLA probes are in proximity, indicated by red-fluorescent dots. Co-localization was present in the cytoplasm.

Recently, CD2-associated protein (CD2AP) and tyrosine kinase substrate with four SH3 domains (TKS4) have emerged as potential cancer formation-regulating factors. These two proteins, which lack enzymatic activity, are scaffolds/adaptors that serve as molecular hubs within cells by binding and releasing multiple signaling molecules via phosphorylation-dependent or -independent interactions mainly mediated by SH3 domains and proline-rich regions (Figure 1A).

TKS4 also serves as a docking site for molecules involved in cell movement and actin rearrangement, e.g., cortactin and N-WASP, and it regulates podosome formation in normal cells and invadopodia formation in cancer cells [8,9]. The involvement of TKS4 in EGF-EGFR-SRC signalling has been previously described, and regulatory roles in mesenchymal stem cell differentiation, adipose tissue, and bone homeostasis have recently been discovered [10,11,12,13]. Recent progress in TKS4-related research has revealed that the effect of TKS4 might also be implicated in several cancer types, including breast cancer [13], melanoma [9], acute myeloid leukemia [14], hepatocellular carcinoma [15] and prostate cancer [16]. Furthermore, TKS4 deficiency has recently been found to disturb EMT-like processes in a colon cancer cell line [17]. 

Although the separate roles of TKS4 and CD2AP in an EMT-like process have been studied, an interaction or co-regulation involving both TKS4 and CD2AP has not been reported. We suspected the possibility of their interplay based on a previous study [18], which demonstrated that TKS4 interacts with a close paralog of CD2AP called CIN85 (SH3KBP1). CD2AP and CIN85 each have three SH3 domains, which are special in the sense that they recognize non-canonical PxP/A/V/IxPR motifs instead of the canonical PxxP SH3 binding sites [19,20] (Figure 1A). CD2AP was originally implicated in dynamic actin remodeling via direct binding to either filamentous actin [21] or cortactin, and is an actin-binding scaffold protein [22]. Later, it was found that CD2AP also plays a regulatory role in epithelial cell junction formation [23], and it was also linked to gastric cancer development as it can inhibit metastasis by promoting cellular adhesion and cytoskeleton assembly [24]. Furthermore, the inhibition of CD2AP by miR-188-5p was associated with poor survival in acute promyelocytic leukemia [25].

To identify potential TKS4-interacting molecules, we conducted a TKS4-IP-MS experiment, which demonstrated that CD2AP is among the TKS4 partner molecules in HCT116 colon cancer cells. We also used ICC colocalization and PLA analysis to confirm the interaction between TKS4 and CD2AP. Furthermore, we applied functional assays to test the interdependent effect of TKS4/CD2AP on oncogenic events. SiRNA-based silencing was used to downregulate their transcript levels and overexpression experiments were performed in HCT116 colon cancer cells to measure the resulting changes in EMT-related gene expression levels and cell migration. 

In this study we demonstrated that CD2AP can bind to TKS4 in vitro and that it co-localizes with TKS4 in HCT116 colon cancer cells. The experimentally induced up and down regulation of the CD2AP and TKS4 expression levels revealed that they both might play a role in tumor development, notably in potentially regulating a partial-EMT state in HCT116 colon cancer cells.

## 2. Results

### 2.1. CD2AP and TKS4 Form a Scaffolding Protein Complex

To shed light on the potential TKS4-CD2AP connection, a high-throughput approach was performed using immunoprecipitation (IP) and mass spectrometry (MS). The identified TKS4-interacting partners, including CD2AP, are shown in Table S1. In this study, we focused on CD2AP-TKS4 interaction in HCT116 colon cancer cells; therefore, we attempted to further confirm the TKS4-CD2AP interaction via IP-Western blot (WB) and immunocytochemistry-based microscopy. The CD2AP-TKS4 interaction was detectable with CD2AP antibody after WB of immunoprecipitated TKS4 (Figure 1B). 

To test the localization of the CD2AP and TKS4 interaction, we performed single and double staining via immunocytochemistry (ICC) (Figure 1C). The individual CD2AP and TKS4 staining combined with actin visualization showed that both proteins are located in the cytoplasm and showed colocalization with actin under the cell membrane. The fluorescence intensity measurement and degree of colocalization computation revealed that the CD2AP/TKS4 double staining showed a high Pearson’s coefficient = 0.817, suggesting substantial colocalization of the two proteins. 

Next, we performed proximity ligation assays (PLAs) to ensure that the apparent colocalization was not an artifact of overlapping signals of two abundant proteins. PLA allows the visualization of the sites within intact cells where the two proteins are in very close proximity (less than 40 nm). The PLA results confirmed the existence of CD2AP-TKS4 complexes under microscopy in fixed cells. (Figure 1D).

The two measurements (ICC double staining and PLA) validate our conclusion that CD2AP/TKS4 are physically colocalized in a protein complex in HCT116 cells.

**Figure 2:**
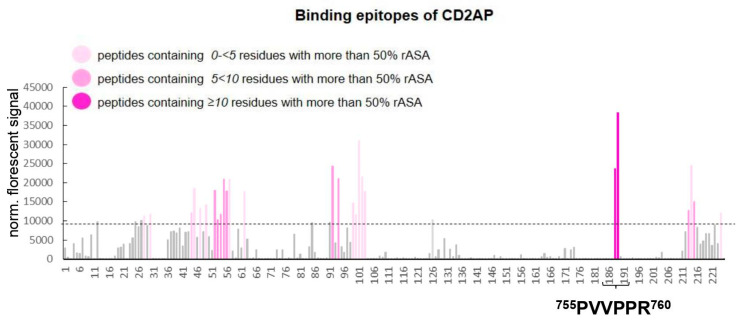
Identification of potential CD2AP binding sites within TKS4. Bars indicate the detected normalized fluorescent signal from the TKS4 peptide microarray, and the dashed line represents the threshold signal intensity (10,000 U). Signals are represented as a mean of three replicates (*n* = 3). The X-axis shows the TKS4 fragments numbered by their positions on the chip. Grey bars represent the normalized fluorescent signals beneath the threshold (10,000 U). The pink shading of the bars indicates the degree of the relative accessible surface area (rASA) value of each 15mer peptide. (Light pink columns represent peptides containing 0–5 AAs with more than 50% rASA value, medium pink columns represent peptides containing 5–10 AAs with more than 50% rASA value, and dark pink columns represent peptides containing ≥10 AAs with more than 50% rASA value.). The PVVPPR motif (located between 755. and 760. AA in the TKS4 protein sequence) is highlighted at the peptides #188 and #189 on the chip.

Next, we decided to map the interaction sites to determine the location of CD2AP-binding to TKS4 by performing a Pepstar Peptide Microarray analysis (Table S2). Assuming that one or more of the folded SH3 domains of CD2AP bind proline-rich short linear motifs in TKS4, the binding of full-length CD2AP protein was screened against fragments covering the entire TKS4 sequence. We employed a chip containing 225 different spots of 15mer peptides derived from TKS4 protein, and during the experiment, we added Myc-tagged CD2AP molecules to the array. The fluorescently labeled anti-Myc-Tag antibody produced a signal only when CD2AP was bound to the TKS4 fragments on the chip. During the evaluation process, we had to exclude false positive signals, which represent the binding of CD2AP to peptide fragments that are hidden structurally deep within the folded TKS4 protein. To evaluate the in vivo accessibility of the peptides within the context of full-length TKS4 protein, we calculated the relative accessible surface area (rASA) values of each amino acid (AA) in the TKS4 protein. High rASA values (100% maximum) indicate that the AAs are located on the surface of folded TKS4 (the AlphaFold2-predicted TKS4 structure was used for calculations); thus, these AAs are accessible for binding partners in intact folded TKS4. The results of microarray analysis showed that peptides 188 and 189 of TKS4 presented the strongest interactions with full-length CD2AP. Peptides 188 and 189 on the chip exhibited the greatest surface accessibility scores (10 of 15 AAs in the peptides have >50% rASA), indicating possible binding sites within TKS4 (Figure 2). Due to the 11-mer overlapping sequences in peptides 188 and 189, both TKS4 peptides contain the known short linear motif ^755^PVVPPR^760^ which is an optimal binding site for the CD2AP SH3 domains (Figure 2). The other TKS4 peptides that bound CD2AP on the chip (indicated with light pink bars in Figure 2) mainly reside within the SH3 domains of TKS4 (see TKS4 structure in Figure 1A). Despite their substantial binding to CD2AP, these peptides exhibit low or medium surface accessibility values; thus, these binding events are probably stemming from domain fragmentation and exposure of their sticky hydrophobic cores (Figure 2, Table S2). Based on our results, we conclude that CD2AP is a novel direct TKS4-interacting partner and that it binds to the ^755^PVVPPR^760^ motif in TKS4 through its SH3 domains. 

### 2.2. Absence of CD2AP and TKS4 Promotes Colon Cancer Cell Migration Separately and Simultaneously via a Partial Epithelial–Mesenchymal Transition Process

Although it has already been reported that TKS4 regulates colon cancer cell migration and plays a role in an EMT-like process [16], CD2AP has also been connected to cancer development and the inhibition of metastasis in gastric cancer (although the underlying mechanisms remain elusive). Therefore, we hypothesized that the TKS4/CD2AP complex cooperatively regulates cytoskeletal rearrangements during cancer progression and participates in EMT process initiation. To investigate the cooperative role of the CD2AP/TKS4 adhesion-related scaffolding proteins, CD2AP and/or TKS4 were silenced via siRNA and cell migration was studied via wound healing assays. Prior to the experiments, the silenced cells’ proliferation was measured via MTT assays, and no changes were observed compared with the control cells (Figure S1).

**Figure 3:**
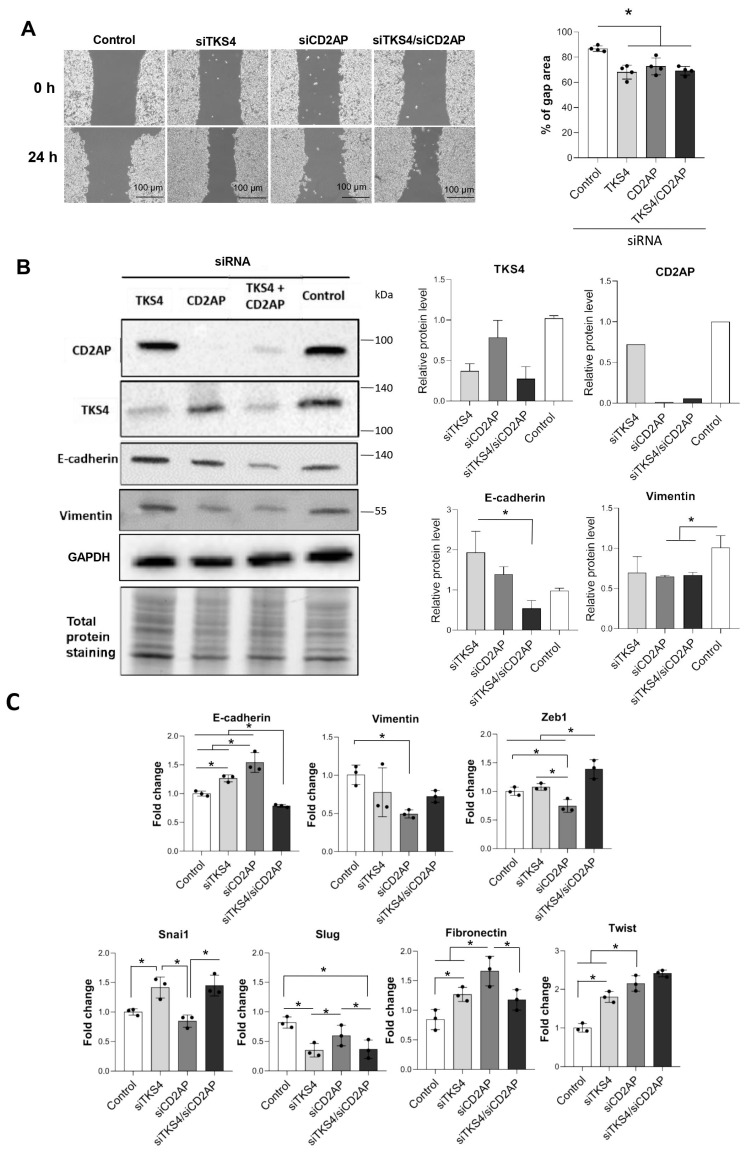
Depletion of *TKS4* and/or *CD2AP* expression in HCT116 colon cancer cells. (**A**) Wound healing assay in *CD2AP-*, *TKS4*-, and *CD2AP/TKS4*-silenced HCT116 cells, and the “% of gap area” was calculated 24 h after scratching. Dots indicate the percent of gap area of three replicates from each group. Between-group comparisons were performed using one-way ANOVA and the Benjamini–Hochberg FDR (false discovery rate) procedure and statistical significance was set at * *p* < 0.05. (*n* = 3); scale bar = 100 µm; gap area (µm). (**B**) EMT marker proteins (E-cadherin and vimentin) level as measured via WB (*n* = 3), validation of efficient CD2AP and TKS4 silencing and densitometry analysis of the marker proteins relative to GAPDH level. Dots indicate the values from three independent experiments. Between-group comparisons were performed using one-way ANOVA and the Benjamini-Hochberg FDR procedure, and statistical significance was set at * *p* < 0.05. (*n* = 3). (**C**) qPCR analyses of EMT markers in si*TKS4*-, si*CD2AP*- and si*CD2AP/TKS4*-double silenced HCT116 cells. Results are shown as fold change relative to the control. Dots indicate the values from three biological replicates. Between-group comparisons were performed using one-way ANOVA and Benjamini–Hochberg FDR procedure, and statistical significance was set at * *p* < 0.05, (*n* = 3).

The results showed that the silencing of CD2AP and/or TKS4 increased colon cancer cell migration compared with the control cells. Simultaneously (CD2AP/TKS4) silenced cells did not show increased migration compared with the single-silenced cells (Figure 3A). To further examine whether increased cell migration stems from an EMT process, we measured the expression levels of EMT-related genes. *Fibronectin* expression increased in the CD2AP-silenced cells (Figure 3C). In TKS4-silenced cells, the protein level of the epithelial marker E-cadherin was increased, which is statistically significant compared with the simultaneously silenced cells but was not different from the control. Vimentin expression was reduced (Figure 3B) upon CD2AP-single and CD2AP/TKS4-double-silencing. The expression level changes in the EMT-related transcription factors (TF) varied between the differently treated cells. *Twist* expression was increased under all silencing conditions. The *Snai1* mRNA levels were elevated, but the *Slug* mRNA levels were decreased in the TKS4-silenced and the CD2AP/TKS4-double-silenced cells. Thus, moderate and diverse expression changes in the EMT-related genes were observed. 

In conclusion, distinct expression patterns of epithelial and mesenchymal markers were detected depending on which genes were silenced.

**Figure 4:**
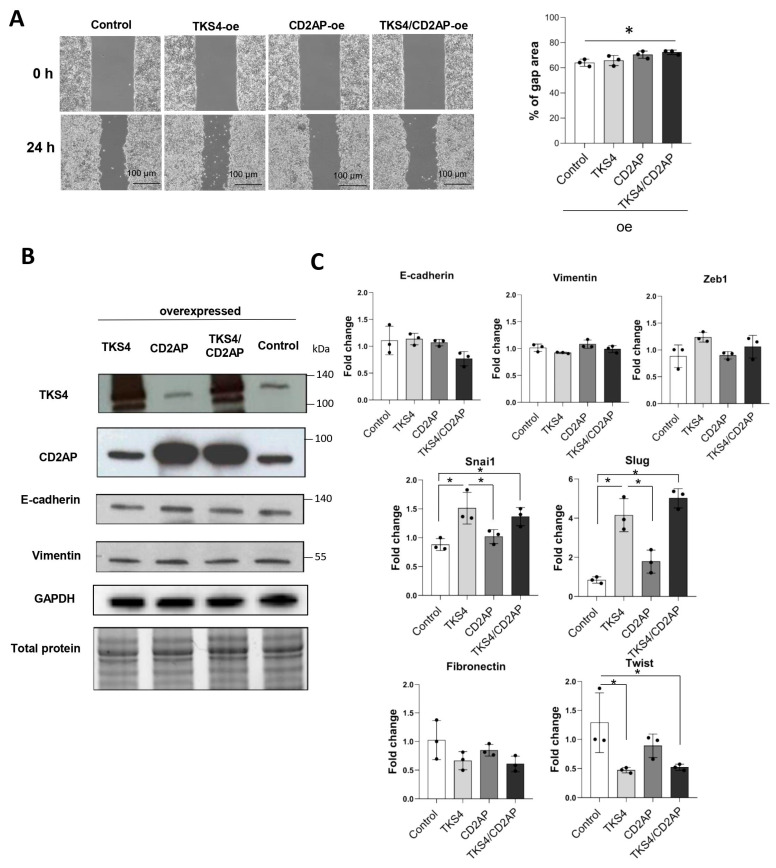
Overexpression of *TKS4* and/or *CD2AP* in HCT116 colon cancer cells. (**A**) Wound healing assays with *CD2AP*-, *TKS4*-, and *CD2AP/TKS4*-overexpressing HCT116 cells and the “% of gap area” was calculated 24 h after scratching. Dots indicate the percentage of gap area of three replicates from each group. Between-group comparisons were performed using one-way ANOVA and the Benjamini–Hochberg FDR (false discovery rate) procedure, and statistical significance was set at * *p* < 0.05. (*n* = 3). (**B**) Protein levels of EMT markers (E-cadherin and vimentin) as measured via WB. Dots indicate the values from three independent experiments. (**C**) qPCR analyses of EMT markers in *TKS4* and/or *CD2AP*- overexpressed (oe) HCT116 cells. Results are shown as fold change relative to the control. Dots indicate the values from three biological replicates. Between-group comparisons were performed using one-way ANOVA and the Benjamini–Hochberg FDR procedure, and statistical significance was set at * *p* < 0.05. (*n* = 3).

To test the hypothesis that CD2AP and TKS4 levels modify cancer cell motility, HCT116 cells were transduced with *CD2AP*- and/or *TKS4*-overexpressing plasmids. The results showed that individual overexpression of *CD2AP* or *TKS4* resulted in a similar level of cell migration compared with control cells; however, simultaneous overexpression of *CD2AP* and *TKS4* caused slightly (albeit significantly) slower cell migration (Figure 4A). We also observed a modest reduction in cell proliferation in TKS4-overexpressing cells and when *CD2AP*/*TKS4*-overexpressing plasmids were used (Figure S1). We confirmed that CD2AP and TKS4 expression were efficiently enhanced after transfection (Figure 4B). 

In *CD2AP*-, *TKS4*-, and *CD2AP/TKS4*-overexpressing HCT116 cells, the expression levels of the main EMT-related markers, i.e., E-cadherin and vimentin, remained at the control level as measured via qRT-PCR and confirmed via WB (Figure 4B,C). Additionally, we measured the mRNA levels of other EMT-related TFs (Figure 4C). We found that the abundance of *Twist* mRNA was decreased, while those of the *Snai1* and *Slug* transcripts were elevated in TKS4-overexpressing and in CD2AP/TKS4-overexpressing cells (Figure 4C). 

## 3. Discussion

In this study, we show that the previously identified gastric cancer metastasis modulating CD2AP adaptor protein also has a role in colon cancer cell motility. In addition, based on IP-MS analysis results, we found that CD2AP and the EMT regulator TKS4 form a protein complex in cells.

The results of the peptide array analysis revealed that CD2AP binds to the ^755^PVVPPR^760^ motif located within the long, disordered linker region between the last two SH3 domains of TKS4 (Figure 1A). The ^755^PVVPPR^760^ sequence in TKS4 represents an optimal recognition motif for the first two SH3 domains of CD2AP (Figure 2). Our results regarding this interaction are reinforced by the results of a previous study that confirmed an interaction between TKS4 and the SH3 domains of the adaptor protein CIN85 [18]. CIN85 is a paralog of CD2AP, and the two proteins show high structural and sequence similarities and their SH3 domains have similar specificity, all recognizing PxP/AxPR motifs [19,20,26]. This implies that the second SH3 domains of both proteins [19,26] and the first SH3 domain of CIN85 [27,28] have also been shown to bind versions of the recognition motif with valine or isoleucine in the middle position. Therefore, we propose that CIN85 likely interacts with TKS4 by binding to the same short linear motif that we identified for CD2AP binding.

We also showed that TKS4 and CD2AP individually and simultaneously modify cell motility and that they might alter EMT-related processes in HCT116 cells. The results of our silencing experiments showed that both epithelial and mesenchymal genes were affected by the reduced TKS4 and CD2AP levels, and cells lacking TKS4 and/or CD2AP also had increased cell motility (Figure 3). Therefore, we propose that decreased TKS4 and CD2AP protein levels can induce a partial-EMT process. 

We manipulated the CD2AP and TKS4 expression levels in the opposite direction via protein overexpression and measured the expression levels of epithelial and the mesenchymal markers and the cell motility-related changes (Figure 4). Cell migration changed only when both proteins were overexpressed and was reduced compared with the control. The levels of key EMT markers, i.e., E-cadherin and vimentin, were not affected by TKS4 and/or CD2AP overexpression (Figure 4B,C). These results indicate that epithelial colon carcinoma cells did not gain a more epithelial phenotype upon the overexpression of TKS4 or CD2AP.

Interestingly, the expression levels of EMT-related TFs, i.e., Snai1 and Slug, were upregulated, while Twist was down regulated upon TKS4 overexpression and in TKS4/CD2AP-double-overexpressing cells. Our results regarding the changes in the expression levels of these EMT-related TFs might seem a bit contradictory since Snai1 expression increased upon TKS4 silencing and overexpression. It is known that Snai1 protein can act as a transcriptional repressor on its own promoter; thus, it can repress its own activity [29]. Therefore, the increased abundance of Snai1 transcript might be due to impaired repression control regulated by TKS4. The fact that Snai1 mRNA level increases both in the absence of TKS4 and when TKS4 is overexpressed suggests that a balance in TKS4 level is needed to maintain the proper Snai1 level. Thus, we hypothesize that TKS4 might have a role in controlling Snai1 transcription.

Our results suggest that as scaffold proteins, TKS4 and CD2AP form a complex that organizes spatially distinct signal transduction pathways by bringing signaling molecules into close proximity. This type of signal control is not unique, as previous studies have also demonstrated that adaptor proteins (e.g., Grb-2, Clb, and Crk) can functionally interact with each other to co-organize the members of different signaling pathways [30,31]. As both TKS4 and CD2AP colocalize with actin (Figure 1C) and bind to cytoskeleton rearranging proteins, we assume that these proteins might cooperatively regulate the actin cytoskeleton and actin-based cell motility processes. It was previously confirmed that TKS4 could be membrane-bound (via the PX-domain) and that it binds and co-localizes with cortactin, an actin-binding protein [8,32,33]. In addition, CD2AP can also interact with cortactin [22,34]. Moreover, it is already known that cortactin promotes colon cancer cell progression [35] and mediates EMT [36]. Therefore, we propose that CD2AP and TKS4 form a stable scaffolding protein complex at the cell membrane and that they both conditionally interact with similar partner molecules as cortactin and SRC as well as distinct CD2AP- and TKS4-specific signaling proteins. This web of interactions could robustly recruit a number of actin-regulating factors to the membrane to induce the actin remodeling required to create membrane protrusions, which are necessary for the formation of membrane ruffles, lamellipodia, or invadopodia to support metastasis. Although the exact mechanism by which this protein complex regulates EMT is not clear, our silencing and overexpression studies show that TKS4 and CD2AP function synergistically during a partial-EMT process. Furthermore, as the two proteins play scaffolding roles in actin remodeling, we propose that the TKS4/CD2AP complex might regulate EMT via actin-based cell motility processes.

Previous studies have shown that molecular interactions between EMT-related proteins can regulate metastasis. It has been proposed that impairing the molecular interactions between adaptor proteins involved in metastasis might be a novel approach for treating aggressive cancer [37]. Similarly, our results revealed that the interaction between CD2AP and TKS4 might have a role in tumor development, particularly in regulating a partial EMT state in colon cancer cells. Thus, targeting this protein–protein interaction might be a novel approach for inhibiting colon cancer metastasis. Further studies are needed to evaluate this hypothesis.

## 4. Materials and Methods

### 4.1. Cell Culture

HCT116 (RRID:CVCL_0291) cells were obtained from ATCC (Manassas, VA, USA) and were tested for mycoplasma contamination using MycoAlert Mycoplasma Detection Kit (Lonza, Basel, Switzerland). HCT116 cells were grown in McCoy’s 5A Medium (Thermo Fisher Scientific, Waltham, MA, USA 16600082) supplemented with 10% FBS (Thermo Fisher Scientific, Waltham, MA, USA, 26400044) and penicillin/streptomycin, and cells were incubated at 37 °C in 5% CO_2_.

### 4.2. siRNA and DNA Plasmid Transfection

Small interfering RNA (siRNA) transfection was performed with Lipofectamine RNAiMax Transfection Reagent (Thermo Fisher Scientific, Waltham, MA, USA, 13778030) according to the manufacturer’s instructions. SiRNAs targeting CD2AP (133808) and TKS4 (252894) were purchased from Thermo Fisher Scientific and used at 10 nM. Scrambled siRNA was used as a negative control at 10 nM. After transfection, cells were incubated for 48 h prior to RNA or protein isolation.

For DNA plasmid transfection, V5-TKS4 and/or MYC-CD2AP (Myc-DDK-Tagged CD2AP clone) (CAT#: RC210191, Origene Technologies Inc., Rockville, MD, USA) plasmids were transfected using X-tremeGENE Hp DNA Transfection Reagent (Roche, Basel, Switzerland) following the manufacturer’s instructions. V5-TKS4 plasmid contained the coding sequence of human TKS4 in the pcDNA3.1/TOPO-V5-His vector [32]. During experiments, negative control wells were treated with transfection reagent only. After transfection, cells were incubated with transfection reagent for 48 h before cell lysis. All experiments were repeated three times.

### 4.3. RNA Isolation and Quantitative Real-Time Polymerase Chain Reaction (qRT-PCR)

RNA was extracted using Direct-zol RNA Miniprep Kit (Zymo Research, Irvine, CA, USA). First Strand cDNA Synthesis Kit (Roche, Basel, Switzerland) and Fast SYBR Green Master Mix (Thermo Fisher Scientific, Waltham, MA, USA) were used for the qRT-PCR (QuantStudioPro6). Data were normalized to the GAPDH and PUM1 transcript levels, and the 2^−ΔΔCt^ method was used to calculate relative gene expression levels. The primers used in this study are listed in Table S3. 

### 4.4. Western Blotting and Immunoprecipitation

For protein extraction, cells were first washed with ice-cold phosphate-buffered saline (PBS). Next, ice-cold harvest buffer (30 mM Tris pH 7.5, with 100 mM NaCl, 1% Triton X-100, 10 mM NaF, 1 mM Na_3_VO_4_, 1 mM EGTA, 2 mM 4-nitrophenyl phosphate, 10 mM benzamidine, 1 mM phenylmethylsulphonyl fluoride, 25 μg/mL Pepstatin A, 25 μg/mL trypsin inhibitor, and 25 μg/mL aprotinin) was added to the cells. Lysates were centrifuged at 14,000 rcf for 10 min at 4 °C. 

Immunoprecipitation was performed using Protein-A Sepharose from *Staphylococcus aureus* (Merck Millipore, Darmstadt, Germany) and anti-TKS4 antibody [30] or without primary antibody in the control group. Briefly, 1.5 mL aliquots of cell lysate, 40 µL of agarose beads, and 10 µL of polyclonal anti-TKS4 antibody were incubated for 1 h at 4 °C and then washed three times with 1 mL PBS + 0.4% Triton-X at 14,000 rcf for 1 min at 4 °C.

Sample loading buffer (4.2 mL H_2_O, 1 mL 6.8 pH Tris, 2.8 mL glycerol, 1.6 mL 10% SDS, 0.4 mL β-mercaptoethanol, 1 mg bromophenol blue) was added to the supernatants, followed by incubation at 95 °C for 5 min. Each protein sample was subjected to 10% SDS-PAGE gel (BioRad, Berkeley, CA, USA) and blotted. Nitrocellulose membranes were blocked and incubated for 60 min with anti-TKS4 ([30], 1:5000) and anti-CD2AP (clone 2A2.1, MABT419, 1:2000) antibodies at room temperature or overnight at 4 °C with anti-E-cadherin (ab15148 1:500), or anti-vimentin (ab137321 1:1000) antibody. After three washes, the membranes were incubated for 60 min with horseradish peroxidase-conjugated secondary antibody (GE Healthcare, Chicago, IL, USA) and then washed four times for 15 min each. The proteins of interest were visualized using enhanced chemiluminescence (ECL) detection reagents (Amersham, Little Chalfont, UK). Chemiluminescent imaging was performed with a ChemiDoc MP system (Bio-Rad, Berkeley, CA, USA). Protein levels were measured relative to GAPDH level with with ImageJ Software (Version 1.53t) [38]. 

### 4.5. Mass Spectrometry Analyses

After immunoprecipitation, samples were loaded onto 10% SDS-PAGE gels. Next, proteins were visualized via Coomassie brilliant blue dye, and gels were fractionated horizontally based on molecular weight (100–300 kDa, 50–100 kDa, 25–50 kDa, and 10–25 kDa). Mass spectrometry analyses were performed via UD-GenoMed Medical Genomic Technologies Kft (Debrecen, Hungary). 

### 4.6. In-Gel Protein Digestion

The protein bands were excised from the gel and subjected to in-gel trypsin digestion. The bands were destained using a 1:1 ratio of 25 mM ammonium bicarbonate (pH 8.5) and 50% acetonitrile followed by the reduction in the proteins using 20 mM dithiothreitol (Sigma, St. Louis, MO, USA) for one hour at 56 °C. The samples were further alkylated with 55 mM iodoacetamide (Sigma, St. Louis, MO, USA) for 45 min in dark. Overnight trypsin digestion was carried out with 100 ng stabilized MS grade trypsin (ABSciex, Framingham, MA, USA) at 37 °C. The reaction was stopped with the addition of concentrated formic acid. The tryptic peptides were extracted from the gel pieces, dried in a vacuum concentrator (Thermo Scientific, Waltham, MA, USA) and kept at −20 °C until the mass spectrometry analysis conducted. 

### 4.7. Liquid Chromatography-Mass Spectrometry Analysis

For protein identification using liquid chromatography with tandem mass spectrometry, the peptides were re-dissolved in 10 μL 1% formic acid (VWR Ltd., Radnor, PA, USA) and were separated in a 180 min water/acetonitrile gradient using an Easy nLC 1200 nano UPLC (Thermo Scientific, Waltham, MA, USA). The peptide mixtures were desalted in an ACQUITY UPLC Symmetry C18 trap column (20 mm × 180 µm, 5 μm particle size, 100 Å pore size; Waters, Milford, MA, USA), followed by separation in a nanoACQUITY Peptide BEH C18 analytical column (150 mm × 75 μm, 1.7 μm particle size, 130 Å pore size; Waters, Milford, MA, USA). The chromatographic separation was performed using a gradient of 5–7% solvent B over 5 min, followed by an increase to 15% of solvent B over 50 min, and then to 35% solvent B over 60 min. Thereafter, solvent B was increased to 40% over 28 min and then to 85% over 5 min, followed by a 10 min increase to 85% of solvent B, after which the system returned to 5% solvent B in 1 min for a 16 min hold-on. Solvent A was 0.1% formic acid in LC water (Sigma, St. Louis, MO, USA); solvent B was 95% acetonitrile (Sigma, St. Louis, MO, USA) containing 0.1% formic acid. The flow rate was set to 300 nL/min. 

Data-dependent acquisition experiments were carried out on an Orbitrap Fusion mass spectrometer (Thermo Scientific, Waltham, MA, USA). The 14 most abundant multiply charged positive ions were selected from each survey MS scan using a scan range of 350–1600 *m*/*z* for MS/MS analyses (Orbitrap analyzer resolution: 60,000, AGC target: 4.0 × 10^5^, acquired in profile mode). Collision-induced dissociation (CID) fragmentation was performed in the linear ion trap with 35% normalized collision energy (AGC target: 2.0 × 10^3^, acquired in centroid mode). Dynamic exclusion was enabled during the cycles (exclusion time: 45 s). 

### 4.8. Protein Identification

The acquired LC-MS/MS data were used for protein identification with the help of MaxQuant 2.0.1 software [39] searching against the Human SwissProt database (release: 2020.06, 20,394 sequence entries) and against the contaminants database provided by the MaxQuant software. Cys carbamidomethylation, Met oxidation and N-terminal acetylation were set as variable modifications. A maximum of two missed cleavage sites were allowed. Results were imported into Scaffold 5.0.1 software (Proteome Software Inc., Portland, OR, USA). Proteins were accepted with at least 2 identified peptides using a 1% protein false discovery rate (FDR) and 95% peptide probability thresholds. 

### 4.9. Cell Proliferation Assay

Cell viability was measured via MTT assays using the Cell Proliferation Kit I (MTT)(Cat. No. 11 465 007 001—Roche, Basel, Switzerland). Briefly, 5 × 10^3^ HCT116 cells were seeded in a 96-well plate in a final volume of 100 μL of culture medium per well. The same day, cells were transfected with siRNAs or plasmids then cell numbers were counted after 24 h. After the appropriate incubation period 10 μL MTT labelling reagent was added to the wells and incubated for 4 h at 37 °C 5% CO_2_. Then, 100 μL Solubilization reagent was added to each well and incubated overnight 37 °C 5% CO_2_. The next day, we measured the absorbance with an ELISA plate reader at 600 nm and a reference absorbance at 700 nm for background measurements.

### 4.10. Wound Healing Assays

HCT116 cells were forward-transfected with siRNAs or plasmid DNA. Next, 5 × 10^5^ cells were cultured in Ibidi Culture-Insert wells (35 mm, Cat. No:81176, Gräfelfing, Germany). After 24 h, the cells reached confluency and the silicone inserts were removed to induce uniform scratches in the cell layer. The dishes were filled with fresh McCoy’s Medium, and the cells were maintained for 24 h to close the wound. Images were acquired using a Leica DMi1 Inverted microscope. The gap areas of each well were calculated using images from each triplicate wells with ImageJ Software (Version 1.53t) [38]. Experiments were repeated three times.

### 4.11. Immunocytochemistry

Cells were fixed with 4% PFA (Thermo Fisher Scientific, Waltham, MA, USA) for 15 min at RT and then permeabilized with 0.1% TritonX-100 in sterile PBS for 10 min at RT, after blocking the samples with 5% BSA and 5% FBS in sterile PBS for 1 h at RT. Next, cells were incubated with the appropriate primary antibody overnight at 4 °C, and after several washing steps, secondary antibodies were used. The cell nuclei were stained with DAPI (Thermo Fisher Scientific). A Zeiss LSM-710 confocal microscopy system (Carl Zeiss Microscopy GmbH, Jena, Germany) was used to detect proteins of interest with a 63× objective. Images were analyzed with ZEN 3.2 (Carl Zeiss Microscopy GmbH, Jena, Germany) and Image J software (Version 1.53t) [38]. Primary antibodies were the same as we used for Western blot. Secondary antibodies used were Goat-Anti-Mouse antibody, Alexa Fluor 488, Thermo Fisher Scientific, Waltham, MA, USA; Cat# A-11029, Goat anti-Rabbit IgG, Alexa Fluor 488, Thermo Fisher Scientific, Waltham, MA, USA; Cat# A-11008, Goat-Anti-Rabbit antibody, Alexa Fluor 546, and Thermo Fisher Scientific, Waltham, MA, USA; Cat# A11035 for ICC analysis. Actin filaments were stained with CF^®^543 Phalloidin (Biotium, Fremont, CA, USA; Cat: 00043). The colocalization coefficient was calculated with Image J-Ezcolocalization plugin according to this article [40]. 

### 4.12. DuoLink Proximity Ligation Assays

HCT116 cells were plated in 12-well Ibidi chambers and cultured for 2 days. Next, the cells were fixed in 4% PFA-PBS and further processed according to the manufacturer’s instructions (DUO92101–1KT, Sigma-Aldrich, Burlington, MA, USA): permeabilization and blocking. The DPLA probe anti-rabbit plus binds to the TKS4 primary antibody (HPA036471, Sigma Aldrich, Burlington, MA, USA, 1:100 dilution), whereas the DPLA probe anti-mouse minus binds to the CD2AP antibody (clone 2A2.1, Millipore, Darmstadt, Germany, 1:250 dilution). The DPLA secondary antibodies generate a signal only when the two DPLA probes interact. Signal detection was conducted with ligation and rolling circle amplification with fluorescently labeled nucleotides. The amplified fluorescent DNA resulted in bright red dots, with each dot representing an individual CD2AP/TKS4 interaction event. Control samples were processed similarly without the addition of the primary antibodies. Staining and image acquisition were performed as previously described [41]. Briefly, nuclei were visualized via DAPI staining (with DuoLink mounting medium), and images were acquired on a Zeiss LSM710 inverted confocal microscope with a 40× objective (Carl Zeiss). For analysis, 5 pictures were taken from each sample type, control samples did not contain red fluorescent colocalization-indicating dots.

### 4.13. Peptide Array

PepStar Peptide Microarray experiments were performed based on the method of Harnoš and colleagues (2018) with some modifications [42]. Microarrays were purchased from JPT (JPT Peptide Technologies GmbH, Berlin, Germany). Peptides (15-mers with 11 overlapping residues) covering the complete TKS4 protein sequence (A1X283) were printed on a glass slide (25 × 75 mm). The peptide microarrays were printed in three identical subarrays. Several controls were also printed on the glass slides to exclude non-specific binding events or to confirm positive signals (Myc-tag, anti-Myc antibody, human serum albumin, human IgG, rabbit IgG). When screening the binding ability of CD2AP to TKS4 peptides, Myc-tagged CD2AP protein was used. Myc-DDK-tagged human CD2-associated protein (RC210191) was purchased from Origene Technologies (Rockville, MD, USA). The screening experiments were performed using two glass slides. As a control, one slide was incubated only with Alexa Fluor^®^ 488-conjugated Myc-Tag (71D10) rabbit monoclonal antibody purchased from CST (Cell Signaling Technology, Danvers, MA, USA). For the measurements, the experimental slides covered with TKS4 fragments were incubated with Myc-CD2AP recombinant protein. The screening experiments and signal visualization were performed via Diagnosticum Zrt (Budapest, Hungary) using a sandwich-like format in a 300 µL reaction volume in an incubation chamber. First, slides were blocked with SmartBlock solution (CANDOR Bioscience, Wangen im Allgäu, Germany, 113,125) for 1 h at 30 °C. Next, the experimental slides were incubated with recombinant Myc-CD2AP protein for 1 h at 30 °C in SmartBlock solution in a final volume of 300 µL. The control slide and the experimental slides were washed four times in 300 ul TBS buffer for 10 min at room temperature then incubated with 1 µg/mL Myc-tag Alexa Fluor 488 antibody in SmartBlock solution in a final volume of 300 µL volume for 1 h at 30 °C. To remove unbound fluorescent antibodies, slides were washed four times with TBS buffer for 10 min at room temperature, and the slides were dried via centrifugation in 50 mL falcon tubes at 1200 rcf for 2 min. Recombinant protein binding was detected by measuring the fluorescence intensity of each peptide spot using a Molecular Devices Axon Genepix 4300A laser scanner. The results were analysed with PepSlide Analyzer Software (version 1.5.8, SICASYS Software GmbH, Germersheim, Germany). The amino acid sequences of the 15-mer TKS4 peptides (with 11 overlapping residues) and the measured fluorescence intensity of the peptide spots on the peptide array are listed in Table S2. The relative accessible surface area (rASA) values of each amino acid of the TKS4 fragments were also calculated. For this, the DSSP method [43] was applied on the AlphaFold2-derived [44] structure of TKS4 and the absolute surface accessibility values provided by DSSP were divided by the respective maximum surface accessibility values of the amino acids as proposed previously [45]. Subsequently, the 15mer TKS4 fragments were classified into three accessibility groups based on the number of their residues with rASA values > 0.5 to evaluate their position relative to the surface of the folded TKS4 protein and thus the possibility that they can be accessed with CD2AP.

### 4.14. Statistical Analyses

Statistically significant differences and between group comparisons were calculated using one-way analysis of variance (ANOVA) analysis followed by the Benjamini–Hochberg procedure to test which subgroup had differing means compared with the control group means [46]. All statistical analyses were performed in GraphPad Prism 8.0.1 (GraphPad Prism, Inc., San Diego, CA, USA).

## 5. Conclusions

Our study revealed that CD2AP is a novel TKS4-interacting protein and together they might form a scaffolding protein complex. The changes in the levels of TKS4 and CD2AP have interdependent regulatory effects on a partial EMT process in colon cancer cells. This protein–protein interaction might represent a novel mechanism by which the signaling leading to a partial-EMT process is modulated. Finally, as the interaction between CD2AP and TKS4 might play a role in tumor development, targeting this protein–protein interaction might be a novel approach for inhibiting cancer metastasis.

## Data Availability

A preliminary version of this manuscript was uploaded to the preprint server BioRxiv: https://www.biorxiv.org/content/10.1101/2023.01.13.523903v4 (accessed on 24 January 2023).

## Supplementary Materials

The following supporting information can be downloaded at: https://www.mdpi.com/article/10.3390/ijms242015136/s1.
