# Supplementary material for: Studying the Association of TKS4 and CD2AP Scaffold Proteins and Their Implications in the Partial Epithelial–Mesenchymal Transition (EMT) Process
Source: Int J Mol Sci. 2023 Oct 13;24(20):15136. doi: 10.3390/ijms242015136 (PMC10606890; doi:10.3390/ijms242015136)

Supplementary Figure S1

Cell proliferation test – siRNA-treated HCT116 cells

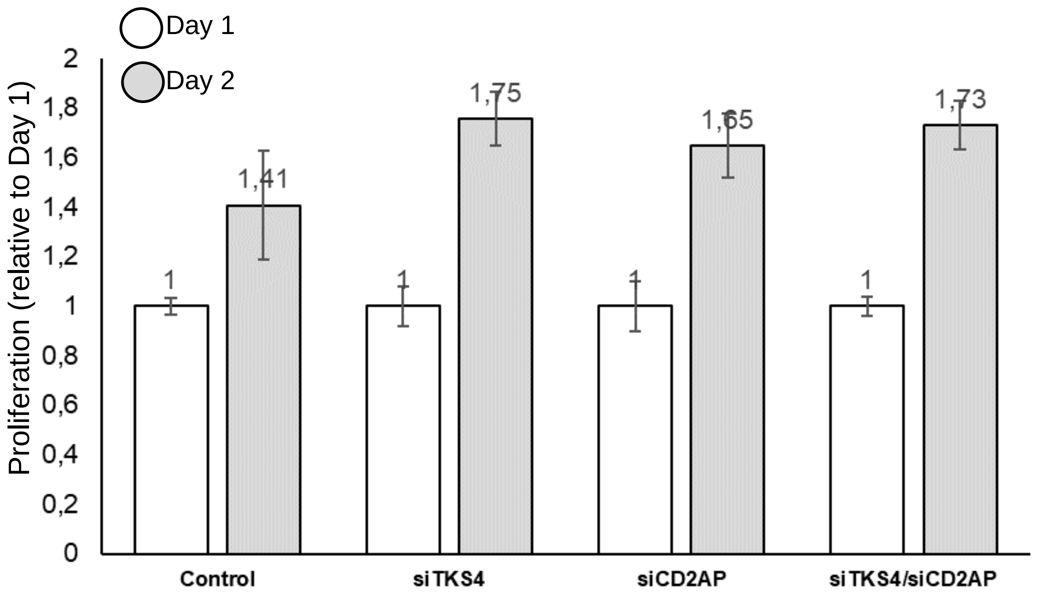

Cell proliferation test – TKS4/CD2AP overexpressed HCT116 cells

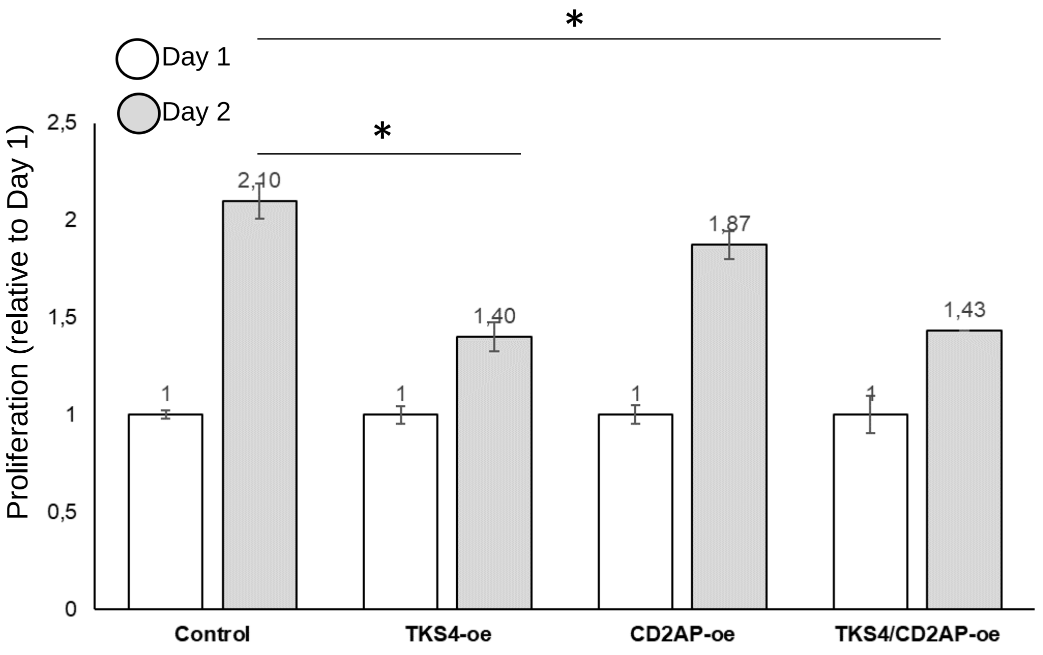

Supplement: Supplementary file 1 [file ijms-24-15136-s001.zip › supp figures_2023_09_06_KA.pdf]
