# Supplementary material for: Studying the Association of TKS4 and CD2AP Scaffold Proteins and Their Implications in the Partial Epithelial–Mesenchymal Transition (EMT) Process
Source: Int J Mol Sci. 2023 Oct 13;24(20):15136. doi: 10.3390/ijms242015136 (PMC10606890; doi:10.3390/ijms242015136)
Supplement: Supplementary file 1 [file ijms-24-15136-s001.zip › supplementary tables_anita_20230728.pdf]

Table S1: List of identified proteins via MS in HCT116 cell lysates immunoprecipitated with anti-TKS4 antibody, Control: HCT116 cell lysates were immunoprecipitated without anti-TKS4 antibody

TKS4 is highlighted with blue, CD2AP is highlighted with yellow

					Unique peptide counts	
	**MS identified Proteins**	**Accession Number**	**Alternate ID**	**MW**	**anti-TKS4-IP sample**	**control sample**
1	SH3 and PX domain-containing protein 2B OS=Homo sapiens OX=9606 GN=SH3PXD2B PE=1 SV=3	SPD2B_HUMAN	SH3PXD2B	102 kDa	16	0
2	Plectin OS=Homo sapiens OX=9606 GN=PLEC PE=1 SV=3	PLEC_HUMAN	PLEC	532 kDa	116	1
3	CD2-associated protein OS=Homo sapiens OX=9606 GN=CD2AP PE=1 SV=1	CD2AP_HUMAN	CD2AP	71 kDa	23	0
4	Cell division cycle and apoptosis regulator protein 1 OS=Homo sapiens OX=9606 GN=CCAR1 PE=1 SV=2	CCAR1_HUMAN	CCAR1	133 kDa	32	0
5	F-actin-capping protein subunit alpha-1 OS=Homo sapiens OX=9606 GN=CAPZA1 PE=1 SV=3	CAZA1_HUMAN	CAPZA1	33 kDa	9	1
6	Tryptophan--tRNA ligase, cytoplasmic OS=Homo sapiens OX=9606 GN=WARS1 PE=1 SV=2	SYWC_HUMAN	WARS1	53 kDa	10	0
7	Kinesin-1 heavy chain OS=Homo sapiens OX=9606 GN=KIF5B PE=1 SV=1	KINH_HUMAN	KIF5B	110 kDa	15	0
8	F-actin-capping protein subunit beta OS=Homo sapiens OX=9606 GN=CAPZB PE=1 SV=4	CAPZB_HUMAN	CAPZB	31 kDa	8	0
9	SH3 domain-binding protein 1 OS=Homo sapiens OX=9606 GN=SH3BP1 PE=1 SV=3	3BP1_HUMAN	SH3BP1	76 kDa	4	0
10	SWISS-PROT:Q3Y5Z3 (Bos taurus) Adiponectin precursor	Q3Y5Z3 (+1)		26 kDa	5	0
11	SH3 domain-containing kinase-binding protein 1 OS=Homo sapiens OX=9606 GN=SH3KBP1 PE=1 SV=2	SH3K1_HUMAN	SH3KBP1	73 kDa	9	0
12	Cytoplasmic dynein 1 heavy chain 1 OS=Homo sapiens OX=9606 GN=DYNC1H1 PE=1 SV=5	DYHC1_HUMAN	DYNC1H1	532 kDa	3	0
13	F-actin-capping protein subunit alpha-2 OS=Homo sapiens OX=9606 GN=CAPZA2 PE=1 SV=3	CAZA2_HUMAN	CAPZA2	33 kDa	3	0
14	Probable ATP-dependent RNA helicase DDX17 OS=Homo sapiens OX=9606 GN=DDX17 PE=1 SV=2	DDX17_HUMAN	DDX17	80 kDa	3	1
15	Polyadenylate-binding protein 4 OS=Homo sapiens OX=9606 GN=PABPC4 PE=1 SV=1	PABP4_HUMAN	PABPC4	71 kDa	4	1
16	Nuclear fragile X mental retardation-interacting protein 2 OS=Homo sapiens OX=9606 GN=NUFIP2 PE=1 SV=1	NUFP2_HUMAN	NUFIP2	76 kDa	3	1
17	ATPase family AAA domain-containing protein 3A OS=Homo sapiens OX=9606 GN=ATAD3A PE=1 SV=2	ATD3A_HUMAN	ATAD3A	71 kDa	4	0
18	60S ribosomal protein L13 OS=Homo sapiens OX=9606 GN=RPL13 PE=1 SV=4	RL13_HUMAN	RPL13	24 kDa	5	2
19	Spermatogenesis-associated serine-rich protein 2 OS=Homo sapiens OX=9606 GN=SPATS2 PE=1 SV=1	SPAS2_HUMAN	SPATS2	60 kDa	4	0
20	Microtubule-associated protein 4 OS=Homo sapiens OX=9606 GN=MAP4 PE=1 SV=3	MAP4_HUMAN	MAP4	121 kDa	6	0
21	60S ribosomal protein L6 OS=Homo sapiens OX=9606 GN=RPL6 PE=1 SV=3	RL6_HUMAN	RPL6	33 kDa	3	0

22	Fibronectin OS=Homo sapiens OX=9606 GN=FN1 PE=1 SV=5	FINC_HUMAN	FN1	272 kDa	6	0

Table S2: Results of the PepStar Peptide Microarray experiments. In this array, 15-amino-acid-long overlapping fragments of Tks4 were spotted on a glass chip and the binding of the fluorescently tagged CD2AP was detected at the interacting sites. Signals are represented as a mean of three replicates (n=3). PX and SH3 domains are shown with bold letters. The relative accessible surface area (rASA) values of each amino acid of the TKS4 fragments were also calculated.

Nr of the spots in the array	Tks4 fragment sequence	Start site in the TKS4 sequence	End site in the TKS4 sequence	Normalized fluorescent signal	location in the domains of TKS4	Nr of residues with >30% rASA	Nr of residues with >50% rASA
1	MPPRRSIVEVKVLDV	1	15	2987,666667		-	-
2	**RSIVEVKVLDVQKRR**	5	19	525	PX domain	-	-
3	**EVKVLDVQKRRVPNK**	9	23	173	PX domain	-	-
4	**LDVQKRRVPNKHYVY**	13	27	4051,333333	PX domain	-	-
5	**KRRVPNKHYVYIIRV**	17	31	1561,666667	PX domain	-	-
6	**PNKHYVYIIRVTWSS**	21	35	1361,333333	PX domain	-	-
7	**YVYIIRVTWSSGSTE**	25	39	5448	PX domain	-	-
8	**IRVTWSSGSTEAIYR**	29	43	723,6666667	PX domain	-	-
9	**WSSGSTEAIYRRYSK**	33	47	668,6666667	PX domain	-	-
10	**STEAIYRRYSKFFDL**	37	51	6294,333333	PX domain	-	-
11	**IYRRYSKFFDLQMQM**	41	55	8	PX domain	-	-
12	**YSKFFDLQMQMLDKF**	45	59	9811,666667	PX domain	-	-
13	**FDLQMQMLDKFPMEG**	49	63	56,33333333	PX domain	-	-
14	**MQMLDKFPMEGGQK D**	53	67	2	PX domain	-	-
15	**DKFPMEGGQKDPKQR**	57	71	2	PX domain	-	-
16	**MEGGQKDPKQRIIPF**	61	75	2	PX domain	-	-
17	**QKDPKQRIIPFLPGK**	65	79	2	PX domain	-	-
18	**KQRIIPFLPGKILFR**	69	83	852	PX domain	-	-
19	**IPFLPGKILFRRSHI**	73	87	2900	PX domain	-	-
20	**PGKILFRRSHIRDVA**	77	91	3125,333333	PX domain	-	-
21	**LFRRSHIRDVAVKRL**	81	95	3930,333333	PX domain	-	-
22	**SHIRDVAVKRLIPID**	85	99	97,33333333	PX domain	-	-
23	**DVAVKRLIPIDEYCK**	89	103	4131	PX domain	-	-
24	**KRLIPIDEYCKALIQ**	93	107	5449,666667	PX domain	-	-
25	**PIDEYCKALIQLPPY**	97	111	9696,333333	PX domain	-	-
26	**YCKALIQLPPYISQC**	101	115	8525,666667	PX domain	-	-
27	**LIQLPPYISQCDEVL**	105	119	10131,33333	PX domain	-	-
28	**PPYISQCDEVLQFFE**	109	123	11305	PX domain	6	3
29	**SQCDEVLQFFETRPE**	113	127	8778,666667	PX domain	-	-
30	**EVLQFFETRPEDLNP**	117	131	11681,66667	PX domain	7	5
31	**FFETRPEDLNPPKEE**	121	135	203,3333333	PX domain	-	-
32	**RPEDLNPPKEEHIGK**	125	139	70,66666667	PX domain	-	-
33	LNPPKEEHIGKKKSG	129	143	2		-	-

34	KEEHIGKKKSGGDQT	133	147	2		-	-
35	IGKKKSGGDQTSVDP	137	151	2		-	-
36	**KSGGDQTSVDPMVLE**	141	155	4966,333333	SH3 1	-	-
37	**DQTSVDPMVLEQYVV**	145	159	7169,666667	SH3 1	-	-
38	**VDPMVLEQYVVVANY**	149	163	7313	SH3 1	-	-
39	**VLEQYVVVANYQKQE**	153	167	6774,666667	SH3 1	-	-
40	**YVVVANYQKQESSEI**	157	171	8059,333333	SH3 1	-	-
41	**ANYQKQESSEISLSV**	161	175	3370	SH3 1	-	-
42	**KQESSEISLSVGQVV**	165	179	7068,333333	SH3 1	-	-
43	**SEISLSVGQVVDIIE**	169	183	7144	SH3 1	-	-
44	**LSVGQVVDIIEKNES**	173	187	12069,66667	SH3 1	7	0
45	**QVVDIIEKNESGWWF**	177	191	18487,66667	SH3 1	4	0
46	**IIEKNESGWWFVSTA**	181	195	5744	SH3 1	-	-
47	**NESGWWFVSTAEEQG**	185	199	13133,66667	SH3 1	4	2
48	**WWFVSTAEEQGWVPA**	189	203	7080,333333	SH3 1	-	-
49	**STAEEQGWVPATCLE**	193	207	14226,33333	SH3 1	3	3
50	**EQGWVPATCLEGQDG**	197	211	5918	SH3 1	-	-
51	VPATCLEGQDGVQDE	201	215	2327,666667		-	-
52	CLEGQDGVQDEFSLQ	205	219	18002,66667		11	8
53	QDGVQDEFSLQPEEE	209	223	10212,66667		15	9
54	**QDEFSLQPEEEEKYT**	213	227	11724,33333	SH3 2	12	6
55	**SLQPEEEEKYTVIYP**	217	231	20852,66667	SH3 2	10	5
56	**EEEEKYTVIYPYTAR**	221	235	17800,33333	SH3 2	8	5
57	**KYTVIYPYTARDQDE**	225	239	20815	SH3 2	8	4
58	**IYPYTARDQDEMNLE**	229	243	2028,666667	SH3 2	-	-
59	**TARDQDEMNLERGAV**	233	247	311,3333333	SH3 2	-	-
60	**QDEMNLERGAVVEVI**	237	251	7725	SH3 2	-	-
61	**NLERGAVVEVIQKNL**	241	255	2916	SH3 2	-	-
62	**GAVVEVIQKNLEGWW**	245	259	17648	SH3 2	3	0
63	**EVIQKNLEGWWKIRY**	249	263	5197,666667	SH3 2	-	-
64	**KNLEGWWKIRYQGKE**	253	267	2	SH3 2	-	-
65	**GWWKIRYQGKEGWAP**	257	271	246	SH3 2	-	-
66	**IRYQGKEGWAPASYL**	261	275	2493,666667	SH3 2	-	-
67	**GKEGWAPASYLKKNS**	265	279	2	SH3 2	-	-
68	**WAPASYLKKNSGEPL**	269	283	2	SH3 2	-	-
69	**SYLKKNSGEPLPPKP**	273	287	2	SH3 2	-	-
70	**KNSGEPLPPKPGPGS**	277	291	2	SH3 2	-	-
71	EPLPPKPGPGSPSHP	281	295	2		-	-
72	PKPGPGSPSHPGALD	285	299	2		-	-
73	PGSPSHPGALDLDGV	289	303	2474		-	-
74	SHPGALDLDGVSRQQ	293	307	2		-	-
75	ALDLDGVSRQQNAVG	297	311	2397		-	-
76	DGVSRQQNAVGREKE	301	315	46,33333333		-	-
77	RQQNAVGREKELLSS	305	319	368,6666667		-	-
78	AVGREKELLSSQRDG	309	323	2		-	-
79	EKELLSSQRDGRFEG	313	327	6436		-	-

80	LSSQRDGRFEGRPVP	317	331	324,3333333		-	-
81	RDGRFEGRPVPDGDA	321	335	1313,666667		-	-
82	FEGRPVPDGDAKQRS	325	339	2		-	-
83	PVPDGDAKQRSPKMR	329	343	2		-	-
84	GDAKQRSPKMRQRPP	333	347	3294		-	-
85	QRSPKMRQRPPPRRD	337	351	9482,666667		-	-
86	KMRQRPPPRRDMTIP	341	355	1697,333333		-	-
87	RPPPRRDMTIPRGLN	345	359	322,3333333		-	-
88	RRDMTIPRGLNLPKP	349	363	162		-	-
89	TIPRGLNLPKPPIPP	353	367	2		-	-
90	**GLNLPKPPIPPQVEE**	357	371	247,6666667	SH3 3	-	-
91	**PKPPIPPQVEEEYYT**	361	375	9667,333333	SH3 3	-	-
92	**IPPQVEEEYYTIAEF**	365	379	24300,66667	SH3 3	10	6
93	**VEEEYYTIAEFQTTI**	369	383	4183	SH3 3	-	-
94	**YYTIAEFQTTIPDGI**	373	387	21021	SH3 3	8	5
95	**AEFQTTIPDGISFQA**	377	391	3174,333333	SH3 3	-	-
96	**TTIPDGISFQAGLKV**	381	395	1804,333333	SH3 3	-	-
97	**DGISFQAGLKVEVIE**	385	399	8070,333333	SH3 3	-	-
98	**FQAGLKVEVIEKNLS**	389	403	4385	SH3 3	-	-
99	**LKVEVIEKNLSGWWY**	393	407	14586,66667	SH3 3	9	2
100	**VIEKNLSGWWYIQIE**	397	411	11530	SH3 3	8	2
101	**NLSGWWYIQIEDKEG**	401	415	30918,33333	SH3 3	8	4
102	**WWYIQIEDKEGWAPA**	405	419	21478,33333	SH3 3	4	3
103	**QIEDKEGWAPATFID**	409	423	17619	SH3 3	6	4
104	**KEGWAPATFIDKYKK**	413	427	2	SH3 3	-	-
105	APATFIDKYKKTSNA	417	431	2		-	-
106	FIDKYKKTSNASRPN	421	435	2		-	-
107	YKKTSNASRPNFLAP	425	439	2		-	-
108	SNASRPNFLAPLPHE	429	443	777		-	-
109	RPNFLAPLPHEVTQL	433	447	421		-	-
110	LAPLPHEVTQLRLGE	437	451	1786,333333		-	-
111	PHEVTQLRLGEAAAL	441	455	160,6666667		-	-
112	TQLRLGEAAALENNT	445	459	48,66666667		-	-
113	LGEAAALENNTGSEA	449	463	2		-	-
114	AALENNTGSEATGPS	453	467	257,3333333		-	-
115	NNTGSEATGPSRPLP	457	471	14,33333333		-	-
116	SEATGPSRPLPDAPH	461	475	313		-	-
117	GPSRPLPDAPHGVMD	465	479	27,33333333		-	-
118	PLPDAPHGVMDSGLP	469	483	2		-	-
119	APHGVMDSGLPWSKD	473	487	2		-	-
120	VMDSGLPWSKDWKGS	477	491	453,3333333		-	-
121	GLPWSKDWKGSKDVL	481	495	55,66666667		-	-
122	SKDWKGSKDVLRKAS	485	499	2		-	-
123	KGSKDVLRKASSDMS	489	503	2		-	-
124	DVLRKASSDMSASAG	493	507	2		-	-
125	KASSDMSASAGYEEI	497	511	1444,333333		-	-

126	DMSASAGYEEISDPD	501	515	10176		-	-
127	SAGYEEISDPDMEEK	505	519	589		-	-
128	EEISDPDMEEKPSLP	509	523	2483,666667		-	-
129	DPDMEEKPSLPPRKE	513	527	2		-	-
130	EEKPSLPPRKESIIK	517	531	5320		-	-
131	SLPPRKESIIKSEGE	521	535	2		-	-
132	RKESIIKSEGELLER	525	539	2509,333333		-	-
133	IIKSEGELLERERER	529	543	559		-	-
134	EGELLERERERQRTE	533	547	3798,666667		-	-
135	LERERERQRTEQLRG	537	551	993		-	-
136	RERQRTEQLRGPTPK	541	555	2		-	-
137	RTEQLRGPTPKPPGV	545	559	2		-	-
138	LRGPTPKPPGVILPM	549	563	2		-	-
139	TPKPPGVILPMMPAK	553	567	260,3333333		-	-
140	PGVILPMMPAKHIPP	557	571	2		-	-
141	LPMMPAKHIPPARDS	561	575	2		-	-
142	PAKHIPPARDSRRPE	565	579	2		-	-
143	IPPARDSRRPEPKPD	569	583	2		-	-
144	RDSRRPEPKPDKSRL	573	587	2		-	-
145	RPEPKPDKSRLFQLK	577	591	2		-	-
146	KPDKSRLFQLKNDMG	581	595	2		-	-
147	SRLFQLKNDMGLECG	585	599	1003		-	-
148	QLKNDMGLECGHKVL	589	603	2		-	-
149	DMGLECGHKVLAKEV	593	607	602,6666667		-	-
150	ECGHKVLAKEVKKPN	597	611	2		-	-
151	KVLAKEVKKPNLRPI	601	615	2		-	-
152	KEVKKPNLRPISKSK	605	619	2		-	-
153	KPNLRPISKSKTDLP	609	623	2		-	-
154	RPISKSKTDLPEEKP	613	627	2		-	-
155	KSKTDLPEEKPDATP	617	631	2		-	-
156	DLPEEKPDATPQNPF	621	635	1075		-	-
157	EKPDATPQNPFLKSR	625	639	2		-	-
158	ATPQNPFLKSRPQVR	629	643	2		-	-
159	NPFLKSRPQVRPKPA	633	647	2		-	-
160	KSRPQVRPKPAPSPK	637	651	2		-	-
161	QVRPKPAPSPKTEPP	641	655	2		-	-
162	KPAPSPKTEPPQGED	645	659	2		-	-
163	SPKTEPPQGEDQVDI	649	663	579,6666667		-	-
164	EPPQGEDQVDICNLR	653	667	1484		-	-
165	GEDQVDICNLRSKLR	657	671	397,6666667		-	-
166	VDICNLRSKLRPAKS	661	675	617		-	-
167	NLRSKLRPAKSQDKS	665	679	2		-	-
168	KLRPAKSQDKSLLDG	669	683	15,66666667		-	-
169	AKSQDKSLLDGEGPQ	673	687	597,3333333		-	-
170	DKSLLDGEGPQAVGG	677	691	2		-	-
171	LDGEGPQAVGGQDVA	681	695	2703		-	-

172	GPQAVGGQDVAFSRS	685	699	26,66666667		-	-
173	VGGQDVAFSRSFLPG	689	703	2419		-	-
174	DVAFSRSFLPGEGPG	693	707	3056,333333		-	-
175	SRSFLPGEGPGRAQD	697	711	2		-	-
176	LPGEGPGRAQDRTGK	701	715	2		-	-
177	GPGRAQDRTGKQDGL	705	719	2		-	-
178	AQDRTGKQDGLSPKE	709	723	2		-	-
179	TGKQDGLSPKEISCR	713	727	2		-	-
180	DGLSPKEISCRAPPR	717	731	2		-	-
181	PKEISCRAPPRPAKT	721	735	57,33333333		-	-
182	SCRAPPRPAKTTDPV	725	739	2		-	-
183	PPRPAKTTDPVSKSV	729	743	2		-	-
184	AKTTDPVSKSVPVPL	733	747	2		-	-
185	DPVSKSVPVPLQEAP	737	751	195,3333333		-	-
186	KSVPVPLQEAPQQRP	741	755	2		-	-
187	VPLQEAPQQRPVVPP	745	759	10,66666667		-	-
188	EAPQQRPVVPPRRPP	749	763	23601		12	10
189	QRPVVPPRRPPPPKK	753	767	38921,66667		12	10
190	VPPRRPPPPKKTSSS	757	771	639		-	-
191	RPPPPKKTSSSSRPL	761	775	53		-	-
192	PKKTSSSSRPLPEVR	765	779	2		-	-
193	SSSSRPLPEVRGPQC	769	783	2		-	-
194	RPLPEVRGPQCEGHE	773	787	272,6666667		-	-
195	EVRGPQCEGHESRAA	777	791	2		-	-
196	PQCEGHESRAAPTPG	781	795	2		-	-
197	GHESRAAPTPGRALL	785	799	2		-	-
198	RAAPTPGRALLVPPK	789	803	2		-	-
199	TPGRALLVPPKAKPF	793	807	2		-	-
200	ALLVPPKAKPFLSNS	797	811	2		-	-
201	PPKAKPFLSNSLGGQ	801	815	2		-	-
202	KPFLSNSLGGQDDTR	805	819	386,3333333		-	-
203	SNSLGGQDDTRGKGS	809	823	247		-	-
204	GGQDDTRGKGSLGPW	813	827	1718,666667		-	-
205	DTRGKGSLGPWGTGK	817	831	42,66666667		-	-
206	KGSLGPWGTGKIGEN	821	835	26		-	-
207	GPWGTGKIGENREKA	825	839	2		-	-
208	TGKIGENREKAAAAS	829	843	2		-	-
209	GENREKAAAASVPNA	833	847	2		-	-
210	EKAAAASVPNADGLK	837	851	2		-	-
211	**AASVPNADGLKDSLY**	841	855	2151,666667	SH3 4	-	-
212	**PNADGLKDSLYVAVA**	845	859	7170,333333	SH3 4	-	-
213	**GLKDSLYVAVADFEG**	849	863	12682,66667	SH3 4	6	5
214	**SLYVAVADFEGDKDT**	853	867	24445,66667	SH3 4	5	4
215	**AVADFEGDKDTSSFQ**	857	871	15045,66667	SH3 4	8	5
216	**FEGDKDTSSFQEGTV**	861	875	8304,333333	SH3 4	-	-
217	**KDTSSFQEGTVFEVR**	865	879	3849	SH3 4	-	-

218	**SFQEGTVFEVREKNS**	869	883	4679,333333	SH3 4	-	-
219	**GTVFEVREKNSSGWW**	873	887	6588	SH3 4	-	-
220	**EVREKNSSGWWFCQV**	877	891	6668,666667	SH3 4	-	-
221	**KNSSGWWFCQVLSGA**	881	895	3578,333333	SH3 4	-	-
222	**GWWFCQVLSGAPSWE**	885	899	9014,666667	SH3 4	-	-
223	**CQVLSGAPSWEGWIP**	889	903	4079,333333	SH3 4	-	-
224	**SGAPSWEGWIPSNYL**	893	907	11970,66667	SH3 4	6	4
225	**SWEGWIPSNYLRKKP**	897	911	61,66666667	SH3 4	-	-

Table S3: List of primers used in RT-qPCR measurements

**Primer name**	**Primer sequence 5'-3'**
GAPDH Forward	TGCACCACCAACTGCTTAGC
GAPDH Reverse	GGCATGGACTGTGGTCATGAG
PUM1 Forward	TGAGTTTATTCCTTCAGACCAGCAG
PUM1 Reverse	GCAAATACCTGTCCCTTAAACGCA
Zeb1 Forward	GGGAGGAGCAGTGAAAGAGA
Zeb1 Reverse	TTTCTTGCCCTTCCTTTCTG
Vimentin Forward	TACAGGAAGCTGCTGGAAGG
Vimentin Reverse	ACCAGAGGGAGTGAATCCAG
E-cadherin Forward	TGCCCAGAAAATGAAAAAGG
E-cadherin Reverse	GTGTATGTGGCAATGCGTTC
TKS4 Forward	GAGGTGAAGGTGCTAGACGT
TKS4 Reverse	GGTCCTTCTGTCCTCCTTCC
CD2AP Forward	AGGAATTCAGCCACATCCAC
CD2AP Reverse	TTGAGGGAAACAGTCCCAAC
